# Human leukocyte telomere length is associated with DNA methylation levels in multiple subtelomeric and imprinted loci

**DOI:** 10.1038/srep04954

**Published:** 2014-05-14

**Authors:** Jessica L. Buxton, Matthew Suderman, Jane J. Pappas, Nada Borghol, Wendy McArdle, Alexandra I. F. Blakemore, Clyde Hertzman, Christine Power, Moshe Szyf, Marcus Pembrey

**Affiliations:** 1 Section of Investigative Medicine, Department of Medicine, Imperial College London, London W12 0NN, UK; 2 University of Bristol, Bristol, UK; 3 McGill University, Montreal, Quebec, Canada; 4 University of Toronto, Toronto, Ontario, Canada; 5 Lebanese International University and Lebanese University, Beirut, Lebanon; 6 University of British Columbia, Vancouver, British Columbia, Canada; 7 UCL Institute of Child Health, London, UK; 8 These authors contributed equally to this work.; 9 Deceased.

## Abstract

In humans, leukocyte telomere length (LTL) is positively correlated with lifespan, and shorter LTL is associated with increased risk of age-related disease. In this study we tested for association between telomere length and methylated cytosine levels. Measurements of mean telomere length and DNA methylation at >450,000 CpG sites were obtained for both blood (N = 24) and EBV-transformed cell-line (N = 36) DNA samples from men aged 44–45 years. We identified 65 gene promoters enriched for CpG sites at which methylation levels are associated with leukocyte telomere length, and 36 gene promoters enriched for CpG sites at which methylation levels are associated with telomere length in DNA from EBV-transformed cell-lines. We observed significant enrichment of positively associated methylated CpG sites in subtelomeric loci (within 4 Mb of the telomere) (*P* < 0.01), and also at loci in imprinted regions (*P* < 0.001). Our results pave the way for further investigations to help elucidate the relationships between telomere length, DNA methylation and gene expression in health and disease.

Telomeres are protective nucleoprotein structures that cap the ends of linear chromosomes. Vertebrate telomeres are composed of variable numbers of a tandem repeat sequence, (TTAGGG)n, bound to the shelterin protein complex^1^. Telomere length is maintained by the action of telomerase in some cell types, notably stem and germ cells. However, in most somatic tissues telomeres shorten with each cell division, a process believed to be accelerated by oxidative stress and inflammation^2^,^3^,^4^,^5^. Very short or dysfunctional telomeres trigger replicative senescence, a process that may be activated by a single critically short telomere in a cell^6^.

In humans, mean leukocyte telomere length (LTL) is positively correlated with lifespan^7^,^8^, and has been proposed as a potential biomarker of biological ageing^9^,^10^,^11^. In support of this hypothesis, mean LTL is generally shorter in adult men than in women at any given age, consistent with shorter average male life expectancy in most populations^12^. Furthermore, numerous studies report associations between LTL and age-related diseases including heart disease, type 2 diabetes (T2D) and cancer^13^,^14^,^15^,^16^, although the causal direction of these relationships remains largely unknown.

There is substantial inter-individual variation in both newborn telomere length and telomere length shortening rates throughout life^17^,^18^. Shorter LTL is associated with several known determinants of ill health, including smoking, excessive alcohol consumption, obesity and chronic life stress such as long-term caring responsibilities or prolonged unemployment^19^,^20^,^21^,^22^,^23^. There is also evidence for association between maltreatment in childhood and shorter adult LTL^24^,^25^, suggesting that early-life exposures may have long-term effects on cellular ageing rates.

In addition to multiple environmental factors, human LTL is influenced by genetic variation. Recent genome-wide association studies (GWAS) have revealed associations between LTL and several common genetic variants^26^,^27^,^28^. Some of the genes within these associated loci have a direct role in telomere maintenance. These include the *TERT* and *TERC* genes, which encode components of the telomerase enzyme, and *OBFC1* and *CTC1*, which encode components of the human CST (Ctc1, Stn1 and Ten1) complex recently shown to inhibit telomerase^29^. Despite their known roles in telomere biology, the effect sizes of individual variants in these loci are small. Importantly however, a genetic risk score analysis showed that inheritance of multiple alleles associated with shorter LTL is associated with an increased risk of coronary artery disease, providing preliminary evidence that telomere shortening might play a causal role in this condition^28^.

The aim of the present study was to further investigate the processes involved in telomere shortening by interrogating epigenetic variation in genomic DNA (specifically methylation of cytosine residues), in order to identify novel loci that are either i) affected by telomere shortening or ii) influence telomere length. Since both telomere length and DNA methylation variations are associated with chronological age^30^,^31^,^32^,^33^, we compared DNA from individuals all aged 44–45 years when blood samples were taken. We first performed HumanMethylation450 microarray analysis and measured telomere length in DNA from male participants in a longitudinal birth cohort. We next investigated the associations between methylation at CpG sites and telomere length in blood DNA. We also performed a parallel investigation in no-passage EBV transformed cell-lines prepared from the same venepuncture, in order to shed light on the potential mechanisms involved in the maintenance of telomere length in this cell type, and to compare them with any identified in blood cells.

## Results

### Telomere length measurements

**Figure 1:**
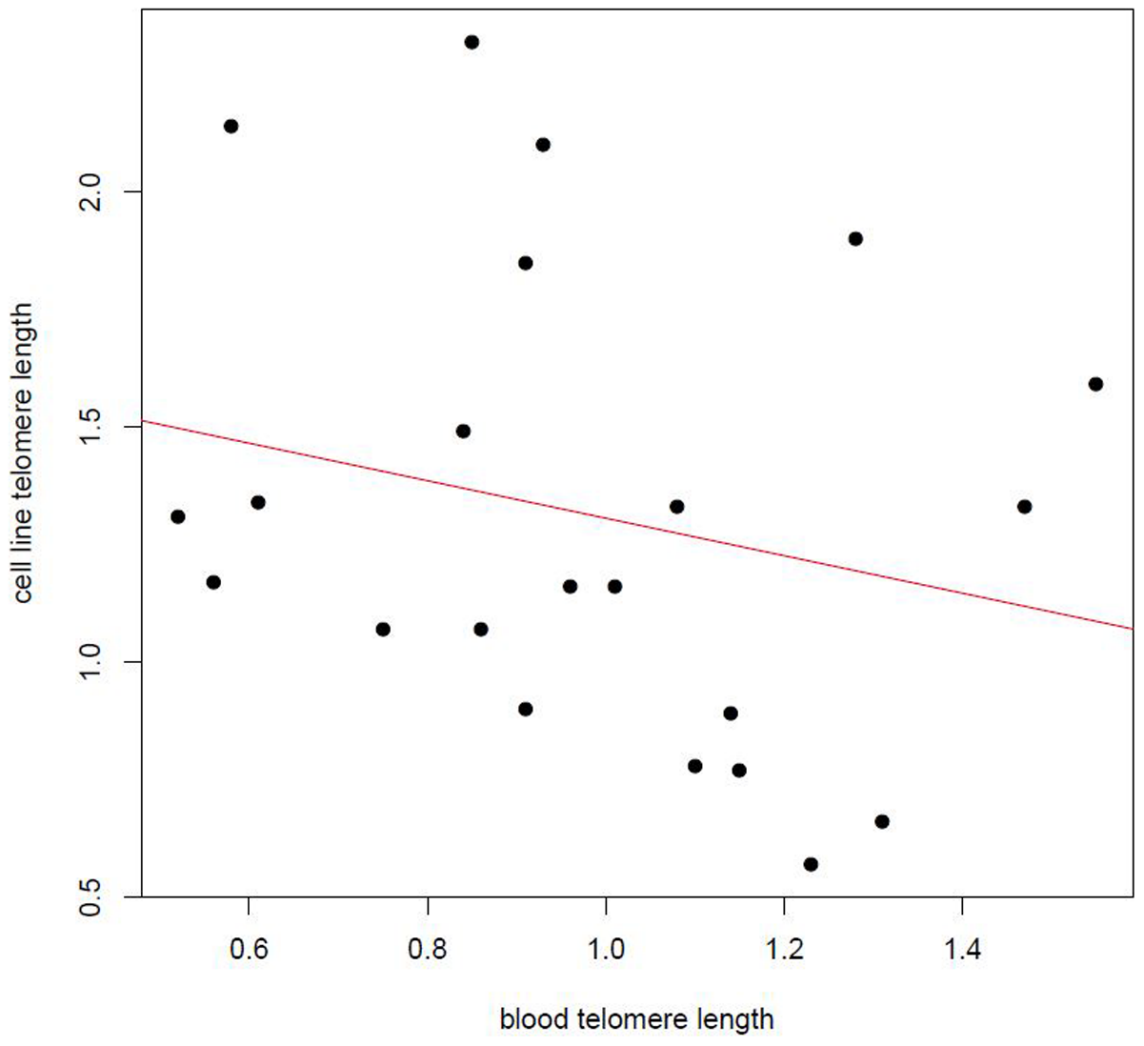
Scatterplot showing lack of correlation between telomere measurements in whole blood and EBV cell-line DNA taken from the same individual (N = 22) (Spearman ρ = −0.3, *P* = 0.18).

Telomere measurements were obtained for 60 DNA samples from 38 different participants. These comprised 24 blood and 36 cell-line samples (for 22 participants, both blood and cell-line DNA was available). Mean relative telomere length measurements were obtained using the multiplex qPCR method^34^, which provides a “T/S ratio” for each DNA sample. This is a relative measure of the amplification of the telomeric DNA sequence (T) compared to that of a single copy gene (S) in each test sample, normalised using a common reference DNA sample. The whole blood telomere measurements were normally distributed (Mean T/S = 1.00, SD = 0.29, range = 0.52–1.55), whereas those obtained for EBV cell-lines were not normally distributed and show a greater range of values (Mean T/S = 1.26, SD = 0.45, range = 0.57–2.32). We found no evidence of correlation between telomere measurements in whole blood and an EBV cell-line DNA taken from the same individual (Spearman ρ = −0.3, *P* = 0.18) (Fig. 1). Six samples showed a decrease in T/S from blood to cell-line; two samples remained same (within 10% of blood T/S value); and 14 samples showed an increase in T/S from blood to cell-line.

### Identification of genes in which DNA methylation is associated with telomere length

In order to identify genes likely to be affected by telomere length-associated methylation, each CpG represented on the microarray was first assigned the Ensembl gene identifier with the nearest transcription start site (TSS), up to a maximum of 10 Kb away. We then identified gene promoters significantly enriched with CpG sites that are individually associated with telomere length (enrichment false discovery rate *P* < 0.05 and at least one CpG site with unadjusted association *P* < 0.05; see Methods for more details).

After applying this stringent approach, we found that overall, association statistics were stronger in whole blood DNA than in cell lines, and were mostly positive, i.e. methylation increases as telomere length increases. We identified 364 sites within 47 different gene promoters that were positively associated with telomere length in whole blood DNA. We also found 100 sites that were negatively associated with telomere length, corresponding to 27 different genes (Table 1 and Data S1). Since some gene promoters contained both positively and negatively associated sites, the total number of gene loci for which methylation levels were associated with telomere length in whole blood was 65.

In cell lines, 94 sites were positively associated with telomere length, corresponding to 28 genes, and 31 sites were negatively associated, corresponding to 12 genes (Table 1 and Data S2). Again, as some promoters contained both positively and negatively associated sites, the total number of gene loci for which methylation levels were associated with telomere length in cell-lines was 36.

**Figure 2:**
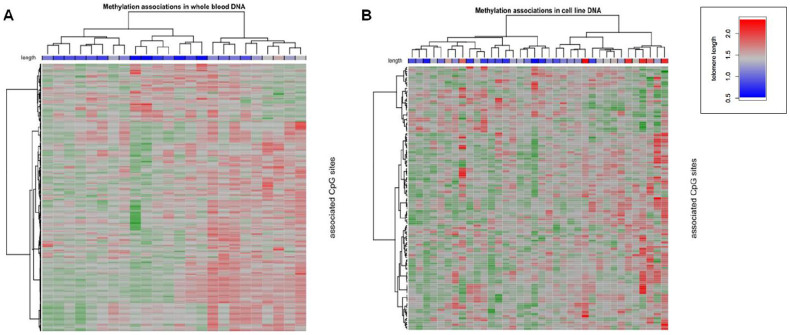
Heatmaps showing M-value variation for CpG sites (rows) across study participants (columns), in A) whole blood and B) cell-line DNA samples. Samples and CpG sites clustered using the Ward algorithm with Pearson's correlation as the distance metric. Relatively lower methylation levels are shown in green and higher methylation levels in red. The key for the telomere length of each sample in both heatmaps is given on the top right of the figure.

The heatmaps in Fig. 2 illustrate these associations, full details of which are given in Data S1 and S2. Together, these results clearly show that methylation is more strongly associated with telomere length in DNA from whole blood than from cell lines.

### Little in common between cell lines and whole blood

There are no individual CpG sites associated with telomere length in both whole blood and cell line DNA, although there are three genes with transcription start sites near sites associated in both cell types: *C15orf26*, *RN5S206* and *L3MBTL1*. Interestingly, the corresponding methylation associations were mostly reversed between the cell types for these three loci. Sites near *C15orf26* were all positively associated with telomere length in cell lines but negatively associated in whole blood cells. Sites near *L3MBTL1* and *RN5S206* tended to be negatively associated with telomere length in cell lines and positively associated in whole blood cells.

### Enrichment for positive associations near telomeres but not centromeres

**Figure 3:**
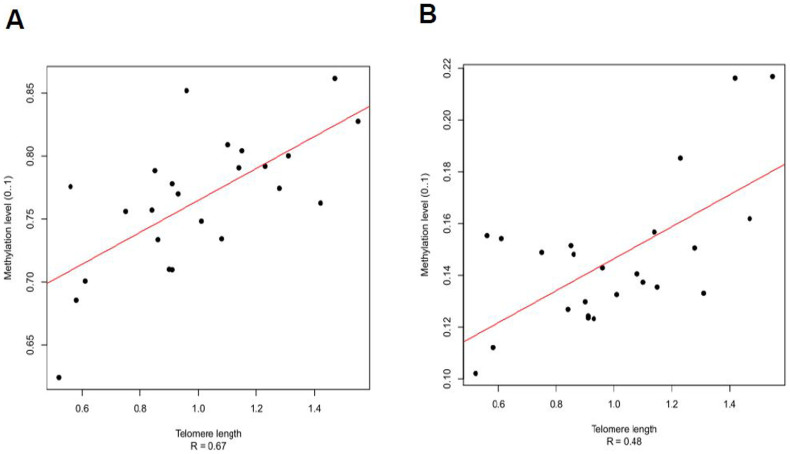
Scatterplots showing examples of positive associations between telomere length (T/S ratio, X-axis) in blood DNA and on the Y-axis, methylation levels at two loci located in subtelomeric regions: A) cg21024264, located 193 kb from chr10 qter within CYP2E1 gene promoter region and B) cg25020933, located 369 kb from chr11 pter within B4GALNT4 gene promoter region. Each point represents the mean T/S ratio of duplicate measurements for an individual DNA sample.

Of the 65 genes enriched for sites associated with telomere length in whole blood, 12 are located near telomeres (within 4 Mb of a chromosome end): *RP11-978I15.10, FAM50B, MAD1L1, CYP2E1, B4GALNT4, DRD4, CEND1, H19, KCNQ1OT1, CREBBP, RP11-1260E13.1* and *ADNP2*. Methylation levels in all but one of the CpG sites in these 12 loci (*RP11-1260E13.1*) are positively associated with telomere length, two examples of which are shown in Fig. 3. This represents a significant enrichment of associated genes located in subtelomeric regions (*P* < 0.01, Fisher's exact test (FET)). In contrast, there is no evidence for any enrichment of associated genes within 4 Mb of centromeres; *P* > 0.85, FET).

We observed nearly identical results in cell lines. Here, enrichment near telomeres was again significant for positively associated sites but not negatively associated sites (*P* = 0.012 and *P* > 0.7, respectively, FET), and not for any associated sites near centromeres (*P* > 0.85, FET).

### Enrichment of imprinted genes

Of the 65 genes with methylation levels associated with telomere length in whole blood DNA (Table 1), 12 are either confirmed (ten) or predicted (two) imprinted human genes, according to information in the geneimprint database (http://www.geneimprint.com, accessed January 2014). For seven of these loci (*MEST, KCNQ1OT1, DIRAS3, FAM50B*, *SGCE*, *L3MBTL1, GNAS-AS1*), only the paternal allele is expressed; for four (*H19, B4GALNT4, HOXA5, MEG3*) only the maternal allele is expressed; and for one (*BLCAP*) parental allele expression is isoform-dependent. Cross-checking of this information using a second database (http://igc.otago.ac.nz/home.html, accessed January 2014) also listed nine of the confirmed imprinted loci present in geneimprint, while the tenth (*DIRAS3*) was listed as provisional. Neither of the two loci listed as ‘predicted’ imprinted loci in geneimprint (*B4GALNT4* and *HOXA5*) were included in the second database. However, since there are only around 80 confirmed and 120 predicted imprinted loci in total in the human genome, the observation of even just ten of these genes in our study represents a highly significant enrichment (*P* < 1 × 10^−5^; permutation test). Four of these imprinted loci (*FAM50B, H19, B4GALNT4 and KCNQ1OT1*) are also located within 4 Mb of a telomere. Moreover, another gene for which methylation levels of nearby CpG sites was associated with telomere length is *ZFP57*, recently shown to be a global regulator of methylation in imprinting control regions (ICRs) in the genome^35^.

### Identification of potential ZFP57 binding sites in silico

Following our discovery that telomere length is associated with methylation levels in both ICRs and the gene encoding a zinc finger protein responsible for their methylation status, *ZFP57*, we hypothesised that Zfp57 may also play a role in the methylation status of subtelomeric regions. We therefore investigated whether subtelomeric regions are enriched for predicted Zfp57 binding sites; a TGCCGC[A/G] sequence in which the cytosine of the CpG is methylated^35^. We identified 45,366 instances of this sequence (irrespective of methylation status) in the human genome (Build hg19/GRCh37) on both the forward and reverse strands. Of those, 8,547 are subtelomeric (within 4 Mb of the end of a chromosome) and 2,913 are centromeric (within 1.5 Mb from the centromere). If uniformly distributed, less than 6,000 motifs would be expected in subtelomeric regions, and more than 4,500 in centromeric regions. Hence, telomeres are enriched and centromeres are depleted of the predicted Zfp57 binding motif (*P* < 2.2 × 10^−16^, hypergeometric). However, since the 450 K array only includes probes for 2,471 of the 45,366 predicted motifs, it was not possible to determine the methylation status of the majority of these target sequences, and thus their likely ability to bind Zfp57 *in vivo*.

### Pathway analysis

Table 2 shows the results of functional and network analyses carried out using the ingenuity pathway analysis system (IPA), for genes in which methylation levels of nearby CpG sites were associated with telomere length. These analyses were carried out separately for the results obtained in both blood and cell-line DNA. Of note, we observed significant enrichment of genes involved in endocrine disorders for the sites associated with telomere length in blood DNA, particularly diabetes mellitus (nine genes, *P* = 3.94e−4) (Table S1). We also observed significant enrichment for imprinting disorders and various forms of cancers. The top canonical pathways enriched for associated loci were ‘dopamine-DARPP32 feedback in cAMP signaling’ in blood (*P* = 8.40e-03) and ‘B cell development’ in cell-line DNA (*P* = 7.61e-04).

## Discussion

Among 44–45 year-old men, we have identified multiple gene promoters enriched for sites at which methylation levels are associated with telomere length in human blood DNA. Furthermore, these associated loci are significantly overrepresented in subtelomeric and imprinted genomic regions. We identified 65 gene promoters that contained sites associated with telomere length, which are largely distinct from the loci in which methylation levels have been associated with increasing age^30^,^31^,^32^,^33^. This lack of overlap is not unexpected, since our study examined telomere length variation at a single chronological age. However, four of the gene promoters that we found to be associated with telomere length also contained sites that belong to a set of 353 ‘age-predictor’ CpGs^30^: those at the *CYP2E1*, *DIRAS3*, *FAM50B* and *SGCE* loci. This may indicate that part of the epigenetic ‘signature’ of chronological age is related to telomere length shortening. It is well-known that there is wide inter-individual variation for the risk of age-related disease in people of the same chronological age. Loci at which methylation levels are associated with both chronological age *and* telomere length may thus be of particular relevance to the investigation of factors that influence successful ageing.

The associations identified in leukocyte DNA may represent either causal, consequential or coincidental relationships, that is, the promoters enriched for associated sites may be: i) genes that encode transcripts involved in regulating telomere length; ii) loci in which epigenetic changes are induced by changes in telomere length or iii) loci in which methylation levels are affected by cellular processes that also influence telomere length. We discuss our results in the context of each of these scenarios.

Several of the 65 loci enriched for CpG sites at which methylation levels are associated with telomere length have potential roles in human telomere biology, and are thus potential regulators of telomere length. Three of the positively-associated sites are within the *MAD1L1* gene, which has been shown in HeLa cells to act as negative regulator of *TERT*, the reverse transcriptase component of the telomerase enzyme^36^. Multiple positively-associated sites are located within the *POU5F1* locus, encoding the key pluripotency transcription factor Oct-4, which regulates expression of the shelterin component TRF1 in mice^37^. We also identified both negative and positively associated sites within the *U1* locus, which encodes part of the spliceosome, but is also essential for processing the RNA component of telomerase in fission yeast^38^. Furthermore, one of the negatively associated sites is located between the *MECOM* and *TERC* genes on chromosome 3; the latter encodes the RNA component of the human telomerase enzyme. Multiple common variants in this locus are associated with leukocyte telomere length in adults^28^. None of the other genes identified through GWAS of telomere length were amongst the loci for which we found methylation associations. However, we did find an association between methylation levels in the *MPL* locus and telomere length. A rare mutation in this gene causes a form of aplastic anaemia, a disorder also caused by mutations in the *TERC* and *TERT* genes^39^.

Associated sites may also be indicative of epigenetic changes induced by telomere length changes. We discovered that a significant proportion of the sites associated with telomere length are located in subtelomeric regions, and that for the majority of these, increased methylation levels are associated with longer telomeres. This finding supports previous evidence from animal and *in vitro* work that heterochromatin is lost in subtelomeric regions as telomeres shorten^40^. Age-related global *hypo*methylation of subtelomeric regions has been observed in both healthy Japanese individuals and those with Parkinson's disease and sarcoidosis^41^,^42^,^43^, although the same group also report *hyper*methylation of subtelomeres in short telomeres in blood DNA from Alzheimer's disease patients^44^. The authors postulate that this latter observation may result from the selective loss of cells with short, hypomethylated telomeres from the blood of Alzheimer's patients. Further longitudinal studies of changes in telomere length and subtelomeric methylation levels in both healthy individuals and those affected by age-related disease are warranted to resolve this issue.

If confirmed, our finding that shorter telomeres are associated with decreased methylation levels of multiple cytosine sites located within 4 Mb of telomeres suggests a possible causal explanation for the relationship between shorter LTL and age-related diseases: as telomeres shorten, the resulting epigenetic changes in subtelomeric regions may alter the expression of disease-related genes. Such a mechanism was first postulated after the discovery of the ‘telomere position effect’ (TPE) in eukaryotic cells, in which the expression of transgenes located close to telomeres is repressed in a telomere length-dependent manner^45^. More recently, investigation of molecular pathology of facioscapulohumeral muscular dystrophy provides a precedent for the involvement of this mechanism in monogenic disease. Symptoms of this condition only appear once telomeres reach a critically short length. Stadler *et al* postulated that the effects of the mutant allele of the candidate gene concerned, *DUX4* (located in 4q35.2), are only apparent once it is “unmasked” by altered expression of this and other genes in subtelomeric regions^46^.

Several of the associated sites we identified in subtelomeric regions are within loci that have a known or potential role in age-related diseases. In particular, two positively associated sites are located within the *CREBBP* locus, which encodes a protein believed to play a central role in the pathogenesis of T2D^47^. Additionally, 15 positively associated sites are located within the *KCNQ1OT1* locus, also involved in T2D susceptibility^48^. Of potential relevance to cardiovascular disease are ten positively associated sites in *ADNP2*, which is involved in the cellular response to oxidative stress^49^.

Finally, we discovered a highly significant enrichment of telomere length-associated methylation sites near imprinted genes. We speculate that this intriguing finding may indicate shared regulation of methylation status of both subtelomeric regions and imprinted genes – potentially via *ZFP57*, a locus in which we identified 21 sites positively associated with telomere length. This gene encodes a global regulator of imprinted genes, part of a specialised version of the KRAB-ZFP/KAP1 system recently been shown to be a general mechanism for establishing methylation patterns in the early mouse embryo. The Zfp57 protein specifically targets only the methylated version of a TGCCGC[A/G] motif in ICRs, ensuring that methylation of the imprinted allele is maintained after fertilisation^35^. We determined that in addition to their expected presence within ICRs, there is enrichment of predicted Zfp57 binding sites in subtelomeric regions. However, only 5.4% of the CpG sites within these predicted target sequences are detected by probes on the 450 K array used in our study. Thus, further investigations are required to determine the methylation status and actual binding of Zfp57 within subtelomeric regions, in DNA isolated from different tissues and developmental stages.

We also investigated telomere length in DNA from EBV-transformed cell lines, which were created using the same venepuncture samples used to prepare the blood DNA samples. We identified multiple sites associated with cell-line telomere length, but there was minimal correlation between these and the sites associated with telomere length in blood DNA. This finding may reflect differences in the regulatory processes involved in controlling telomere length in somatic cells versus those involved in maintaining telomere length in immortalised cells. Telomere length in germ cells, haematopoietic and other stem cells is maintained by the action of telomerase. However, a telomerase-independent pathway, known as “alternative lengthening of telomeres” (ALT), is activated in some cancer cells. There is also some evidence for activation of ALT in cells newly transformed with EBV^50^. This pathway synthesises telomeric sequences independently of telomerase, using homologous recombination following telomere sister chromatid exchange. Our results are consistent with distinct mechanisms being responsible for maintaining telomere length in blood and early passage EBV-transformed cells, with activation of the ALT pathway in transformed cells overriding DNA methylation states of genes that are associated with telomere length and telomerase activity in blood cells. Some of the CpG sites identified in our study as being associated with telomere length in cell-line DNA may provide further insights into the immortalisation process and the consequences of EBV infection.

Our study of methylation and telomere length was based on a relatively small number of samples, taken at one time point. Additionally, we only studied telomere length in blood DNA, so the relevance of our findings to other tissues remains to be determined. However, the LTL measurement method gives a mean value for the telomeres of all chromosomes in all the different types of leukocyte present in blood, which in turn is presumed to reflect telomere length of the haematopoietic stem cells. It is likely that mechanisms that regulate telomere length are common to all healthy somatic tissues and the stem cells from which they are derived, since there is high correlation between telomere length in multiple tissues from the same individuals^51^.

One strength of our study is the homogenous nature of the population with respect to age and gender (i.e. all men aged 44–45 at the time blood samples were taken). Accordingly, the potential confounding effects of these variables - both known to be strongly associated with telomere length and DNA methylation - were absent from our analyses. A further consequence of the age group examined is that our findings represent methylation associations with telomere length, rather than chronological age, and are thus of relevance to the study of healthy ageing.

The results of our pilot study suggest that as telomeres shorten, the methylation levels of many gene promoters in subtelomeric regions may change, which in turn could cause changes in gene expression that increase the risk of age-related disease. Since LTL is highly heritable, this supports the notion that ‘telotype’ may contribute towards explaining some of the missing heritability observed for many common conditions with a genetic component, as proposed by Armanios and Blackburn^52^. Further functional and epidemiological investigations of these observations are required to confirm the role of telomere length-dependent gene expression in health and disease.

## Methods

### Samples

Genomic DNA samples from both whole blood and EBV cell-lines from adult males aged 44–45, participants in the 1958 British Birth Cohort Study, were available for this study^53^. Details of these samples have been reported previously^54^, and the characteristics of the 38 participants included in this study are summarised in Table 3. In brief, 40 male participants were originally selected to represent extremes of socioeconomic position in child and adulthood, and other characteristics namely, *in utero* exposure to tobacco and childhood abuse. Telomere length measurements and DNA methylation data were obtained using the same DNA sample for 38 of the original 40 males (those for whom enough blood or cell-line DNA was available for both these analyses). DNA from whole blood was available for 24 males and from EBV cell lines N = 36, while for 22 participants both whole blood and EBV cell line DNA was available.

### Ethics statement

All participants provided written consent and a blood sample for DNA analysis and EBV-transformation into a lymphoblastoid cell-line for future studies. Ethical approval was given by the South-East Multi-Centre Research Ethics Committee. All methods were carried out in accordance with approved guidelines.

### Telomere measurements

Mean relative telomere length was measured in genomic DNA samples prepared from either whole blood or EBV cell-lines, using a multiplex quantitative real-time PCR method^34^ with minor modifications as described previously^22^. To minimise intra-assay variability, all PCRs were carried out on a single 384-well plate on a CFX384 Real-time PCR detection system (Bio-Rad). Five serial dilutions of a reference sample (leukocyte DNA from a 42-year-old non-cohort female) spanning 5–50 ng were run in triplicate, in addition to a no-template control (NTC). Human beta-globin (Hgb) was used as the single copy reference gene. Following amplification and data collection, the CFX manager software (Bio-rad) was used to generate standard curves for the reference DNA dilutions, one for the telomere signal (T) and one for the single copy gene signal (S). Telomere measurements for each sample were calculated as T/S ratios, a relative measure of the amplification of the telomeric DNA sequence compared to that of the single copy gene. The T/S values reported are the mean of duplicate measurements for each sample, and the overall mean coefficient of variation (CV) between duplicates was 4%.

#### Infinium HumanMethylation450 microarray analysis

DNA methylation profiles were generated from the DNA of whole blood samples and EBV cell lines using the Infinium HumanMethylation450 microarray^55^. In all, there were 22 study participants with both a whole blood DNA methylation profile as well as an EBV cell line methylation profile.

Microarray quality was assessed by generating quality control plots using the minfi Bioconductor package and found to be satisfactory^56^. Microarrays were background corrected and normalised to control probes using the minfi package. The probe intensities were then transformed into beta-values (M/(M + U)) where M and U are the corresponding methylated, and unmethylated, probe intensities, respectively. Although beta-values can be conveniently interpreted as methylation levels, association analyses were carried out using so-called M-values, logit-transformed beta values as previously recommended^57^.

Statistically significant associations between DNA methylation and telomere length were identified by fitting linear models using the limma Bioconductor package^58^ with microarray M-values as the dependent variables and sample telomere lengths as the independent. In addition, models included adjustments for potential confounding due to technical artifacts such as plate effects, as well as individual characteristics including childhood and adulthood SEP, childhood abuse and maternal smoking during pregnancy. Adjustments were made by including independent surrogate variables in the limma design matrix as identified by the DoISVA function of the isva R package^59^. The DoISVA function was applied to the M-values twice, once with no potential confounders included in the input and a second time with the technical and individual variables noted above along with any statistically significant variables identified in the first call to DoISVA (False discovery rate (FDR) < 0.05). The resulting two most statistically significant independent surrogate variables were then retained for inclusion in the limma design matrix.

In order to identify genes likely to be affected by telomere length-associated methylation, each CpG represented on the microarray was assigned the Ensembl gene identifier with the nearest TSS not more than 10 Kb away (retrieved January 2013). The Wilcoxon rank-sum test was then applied to compare the limma t-statistics of the CpG sites associated with each gene to the limma t-statistics of the CpG sites not associated with the gene. Hence, a significant *P*-value indicates that the CpG sites associated with the gene are enriched for methylation levels either positively or negatively correlated with telomere length. FDRs were calculated from these *P*-values in order to control for multiple testing using the Benjamini-Hochberg algorithm. The methylation levels of an individual CpG site were considered significantly associated with telomere length if the FDR of its associated gene was <0.05 (‘Q value’) and if the unadjusted *P* value for the site calculated by limma was <0.05. All such sites are listed in Data S1 and S2.

### Enrichment of sites near imprinted genes

Given how methylation associations are identified, it is not possible to simply apply Fisher's exact test to determine whether there is a surprisingly large number of imprinted genes near significantly associated CpG sites. This is because genes near a large number of CpG sites represented on the microarray are more likely to be near a significantly associated CpG site than another gene near a small number of CpG sites. We confirmed that this was the case for imprinted genes, which are on average near twice as many microarray CpG sites as expected. Consequently, we designed a permutation test that adjusts for this bias. We first identified the nearest gene TSS for each CpG site and then calculated for each gene the number of such CpG sites, called the *nearest count* of the gene. For our permutation test, we repeatedly selected (100,000 times) gene sets with the same size and “nearest count” distribution as the set of genes found significantly associated with telomere length. Whereas our associated gene set contained 12 imprinted genes, the average random gene set contained only 1 imprinted gene, and only one random gene set contained 10 imprinted genes.

### Pathway analysis

Functional and network relationships between the genes associated with CpG sites in which methylation levels are correlated with telomere length were investigated using the Ingenuity Pathway Analysis (IPA) software tool (Ingenuity Systems, www.ingenuity.com).

## Supplementary Material

Supplementary Information Data S1

Supplementary Information Data S2

Supplementary Information Table S1

## Figures and Tables

**Table 1:** Identification of gene promoters enriched for sites at which methylation levels are associated with telomere length: summary data (SD = standard deviation, TSS = transcription start site)

	Blood (N = 24)	Cell-line (N = 36)
Mean telomere length, T/S ratio (SD)	1.00 (0.29)	1.26 (0.45)
Genes with TSS near associated CpG sites	65	36
Imprinted genes with associated CpG sites	12	3
Genes near telomeres with associated CpG sites	12	8
Associated CpG sites (all)	464	125
Associated CpG sites (positive)	364 (78%)	94 (75%)
Associated CpG sites (negative)	100 (22%)	31 (25%)

**Table 2:** Results of Ingenuity pathway and network analysis for genes with promoters enriched for CpG sites at which methylation levels are associated with telomere length in either blood DNA (65 genes) or cell-line DNA (36 genes). Details of the disorders and gene names for the analysis of blood DNA are given in Table S1

BLOOD DNA			CELL LINE DNA		
**DISEASES AND DISORDERS**					
**Name**	***P*-value**	**No. genes**	**Name**	***P*-value**	**No. genes**
Developmental Disorders	9.59E-05 - 4.39E-02	13	Developmental Disorders	1.28E-03 - 4.40E-02	4
Endocrine System Disorders	9.59E-05 - 4.39E-02	18	Endocrine System Disorders	1.28E-03 - 3.91E-02	3
Gastrointestinal Disease	9.59E-05 - 4.77E-02	15	Hereditary Disorders	1.28E-03 - 4.77E-02	4
Hereditary Disorders	9.59E-05 - 4.39E-02	15	Infectious Disease	1.28E-03 - 4.41E-02	6
Reproductive System Disease	9.59E-05 - 4.22E-02	14	Metabolic Disease	1.28E-03 - 3.91E-02	2
**MOLECULAR AND CELLULAR FUNCTION**					
**Name**	***P*-value**	**No. genes**	**Name**	***P*-value**	**No. genes**
Gene Expression	1.86E-06 - 4.77E-02	19	Cell Cycle	1.28E-03 - 3.66E-02	2
Cellular Development	1.39E-04 - 4.77E-02	16	Cellular Compromise	1.28E-03 - 1.28E-03	1
Cell Morphology	6.62E-04 - 3.68E-02	12	Cellular Development	1.28E-03 - 4.52E-02	8
Cell-To-Cell Signaling and Interaction	1.07E-03 - 3.78E-02	10	Cellular Movement	1.28E-03 - 3.04E-02	4
Drug Metabolism	1.07E-03 - 3.78E-02	3	Energy Production	1.28E-03 - 1.02E-02	2
**PHYSIOLOGICAL PUBLIC "-//NPG//DTD XML Article//EN" DEVELOPMENT AND FUNCTION**					
**Name**	***P*-value**	**No. genes**	**Name**	***P*-value**	**No. genes**
Connective Tissue Dev't and Function	2.14E-05 - 4.03E-02	7	Cardiovascular System Development and Function	1.28E-03 - 4.15E-02	2
Embryonic Development	2.14E-05 - 4.81E-02	15	Embryonic Development	1.28E-03 - 4.52E-02	3
Organ Development	2.14E-05 - 4.52E-02	14	Nervous System Development and Function	1.28E-03 - 4.28E-02	3
Organ Morphology	2.14E-05 - 4.52E-02	12	Organ Development	1.28E-03 - 4.52E-02	3
Organismal Development	2.14E-05 - 4.81E-02	13	Organ Morphology	1.28E-03 - 4.89E-02	6
**TOP CANONICAL PATHWAYS**					
**Name**	***P*-value**	**Ratio**	**Name**	***P*-value**	**Ratio**
Dopamine-DARPP32 Feedback in cAMP Signaling	8.40E-03	3 of 186	B Cell Development	7.61E-04	2 of 33
Lymphotoxin b Receptor Signaling	8.46E-03	2 of 61	Nur77 Signaling in T Lymphocytes	2.24E-03	2 of 63
Prod'n of NO and ROS in Macrophages	1.08E-02	3 of 211	IL-4 Signaling	4.01E-03	2 of 79
Pregnenolone Biosynthesis	1.53E-02	1 of 13	SAPK/JNK Signaling	6.53E-03	2 of 103
Dopamine Receptor Signaling	1.63E-02	2 of 95	iCOS-iCOSL Signaling in T Helper Cells	8.21E-03	2 of 123

**Table 3:** Characteristics of the 38 male study participants

	Age (y)		n
Childhood manual social class n (%)	0	19 (50.0)	38
Adulthood manual social class, n (%)	42	20 (52.6)	38
Education level – below O level, n (%)	42	9 (23.7)	38
Smoker, n (%)	42	10 (27.0)	37
Alcohol drinks daily, n (%)	42	9 (24.3)	37
Birth weight, g, mean ± SD	birth	3522 (590)	37
Height, m, mean ± SD	7	1.23 (0.07)	35
Height, m, mean ± SD	42	1.77 (0.08)	37
Body mass index, kg/m^2^, mean ± SD	45	27.5 (4.07)	38
Waist circumference, cm, mean ± SD	45	99.6 (10.5)	38
Systolic blood pressure, mmHg, mean ± SD	45	134.4 (18.3)	38
Diastolic blood pressure, mmHg, mean ± SD	45	84.5 (11.6)	38
FEV1*, mean ± SD	45	3.82 (0.64)	34

*FEV1 = one-second forced expiratory volume; best test of three spirometry readings.
