# Supplementary material for: Human leukocyte telomere length is associated with DNA methylation levels in multiple subtelomeric and imprinted loci
Source: Sci Rep. 2014 May 14;4:4954. doi: 10.1038/srep04954 (PMC4344300; doi:10.1038/srep04954)
Supplement: Supplementary Information — Table S1 [file srep04954-s3.pdf]

Supplementary Table S1

Human leukocyte telomere length is associated with DNA methylation levels in multiple subtelomeric and imprinted loci

Jessica L. Buxton, Matthew Suderman, Jane Pappas, Nada Borghol, Wendy McArdle, Alexandra I.F. Blakemore, Clyde Hertzman, Christine Power, Moshe Szyf and Marcus Pembrey.

TABLE S1

Full results of ‘Diseases and Disorders’ Ingenuity pathway and network analysis for genes with promoters enriched for CpG sites at which methylation levels are associated with telomere length in blood DNA (65 genes).

**Name of disease/disorder**	***P*-Value**	**Genes**	**No. of genes**
**Developmental Disorders**			
Beckwith-Wiedemann syndrome	9.59E-05	H19,KCNQ1OT1	2
overgrowth syndrome	3.57E-04	H19,KCNQ1OT1,RNF135	3
Russell-Silver syndrome	2.57E-03	H19	1

atresia of oocytes	2.57E-03	FOXL2	1
combined oxidative phosphorylation deficiency 2	2.57E-03	MRPS16	1
congenital amegakaryocytic thrombocytopenia	2.57E-03	MPL	1
macrocephaly, macrosomia, facial dysmorphism syndrome	2.57E-03	RNF135	1
type 1 blepharophimosis, epicanthus inversus, and ptosis	2.57E-03	FOXL2	1
type 2 blepharophimosis, epicanthus inversus, and ptosis	2.57E-03	FOXL2	1
Ehlers-Danlos syndrome type III	5.13E-03	TNXB	1
Rubinstein-Taybi syndrome	5.13E-03	CREBBP	1

dysplasia of placental tissue	5.13E-03	H19	1
disorder of stature	6.09E-03	H19,HOXA5,KCNQ1OT1	3
bare lymphocyte syndrome, type i	7.68E-03	TAPBP	1
pseudohypoparathyroidism type 1B	7.68E-03	GNAS-AS1	1
aphasia	1.78E-02	DRD4	1
multiple congenital anomalies	2.63E-02	CREBBP,FOXL2,H19,KCNQ1OT1	4
congenital anomaly of musculoskeletal system	2.87E-02	CREBBP,H19,mir-10,RNF135,TNXB	5
dysplasia	2.95E-02	H19,MRPS16,TNXB	3
malformation of brain	3.52E-02	CREBBP,MRPS16,RNF135	3
dwarfism	4.39E-02	H19,HOXA5	2
**Endocrine System Disorders**			

Beckwith-Wiedemann syndrome	9.59E-05	H19,KCNQ1OT1	2
diabetes mellitus	3.94E-04	CYP2E1,DRD4,mir-10,NR4A2,POU5F1,PPP1R11,TNXB,TRIM31,ZFP57	9
insulin-dependent diabetes mellitus	1.26E-03	POU5F1,PPP1R11,TNXB,TRIM31,ZFP57	5
pituitary dysfunction	1.34E-03	DRD4,H19,KCNQ1OT1	3
type 1 blepharophimosis, epicanthus inversus, and ptosis	2.57E-03	FOXL2	1
transitory neonatal diabetes	5.13E-03	ZFP57	1
pseudohypoparathyroidism type 1B	7.68E-03	GNAS-AS1	1
thyroid cancer	1.20E-02	DIRAS3,LTB,POU5F1	3
hyperprolactinemia	3.53E-02	DRD4	1
chronic pancreatitis	3.78E-02	CYP2E1	1
ovarian cancer	4.22E-02	DIRAS3,H19,HOXA5,MECOM,PRDM9	5

metabolic syndrome X	4.39E-02	CYP2E1,DRD4	2
**Gastrointestinal Disease**			
Beckwith-Wiedemann syndrome	9.59E-05	H19,KCNQ1OT1	2
diabetes mellitus	3.94E-04	CYP2E1,DRD4,mir-10,NR4A2,POU5F1,PPP1R11,TNXB,TRIM31,ZFP57	9
insulin-dependent diabetes mellitus	1.26E-03	POU5F1,PPP1R11,TNXB,TRIM31,ZFP57	5
transitory neonatal diabetes	5.13E-03	ZFP57	1
tumorigenesis of hepatocellular carcinoma	9.71E-03	LTB,MAD1L1	2
formation of aberrant crypt foci	1.78E-02	CYP2E1	1
meteorism	2.29E-02	HOXA5	1
chronic hepatitis	2.47E-02	LTB,MPL	2
formation of intestinal polyp	3.04E-02	H19	1

incidence of hepatocellular carcinoma	3.04E-02	LTB	1
inflammation of liver	3.24E-02	CYP2E1,LTB,MPL	3
chronic pancreatitis	3.78E-02	CYP2E1	1
colonic polyposis	4.77E-02	H19	1
**Hereditary Disorders**			
Beckwith-Wiedemann syndrome	9.59E-05	H19,KCNQ1OT1	2
Russell-Silver syndrome	2.57E-03	H19	1
combined oxidative phosphorylation deficiency 2	2.57E-03	MRPS16	1
congenital amegakaryocytic thrombocytopenia	2.57E-03	MPL	1
macrocephaly, macrosomia, facial	2.57E-03	RNF135	1

dysmorphism syndrome			
recurrent hydatidiform mole type 2	2.57E-03	KHDC3L	1
tenascin-X-deficiency	2.57E-03	TNXB	1
type 1 blepharophimosis, epicanthus inversus, and ptosis	2.57E-03	FOXL2	1
type 2 blepharophimosis, epicanthus inversus, and ptosis	2.57E-03	FOXL2	1
Ehlers-Danlos syndrome type III	5.13E-03	TNXB	1
myoclonic dystonia	5.13E-03	SGCE	1
transitory neonatal diabetes	5.13E-03	ZFP57	1
PTEN hamartoma tumor syndrome	7.68E-03	KLLN	1
bare lymphocyte syndrome, type i	7.68E-03	TAPBP	1

pseudohypoparathyroidism type 1B	7.68E-03	GNAS-AS1	1
familial Parkinson disease	3.53E-02	NR4A2	1
dwarfism	4.39E-02	H19,HOXA5	2
**Reproductive System Disease**			
Beckwith-Wiedemann syndrome	9.59E-05	H19,KCNQ1OT1	2
genital tumor	3.60E-04	B4GALNT4,DIRAS3,FOXL2,H19,HOXA5,KHDC3L,MAD1L1,MECOM,mir-10,mir-7,POU5F1,PRDM9	12
pituitary dysfunction	1.34E-03	DRD4,H19,KCNQ1OT1	3
Sertoli-Leydig cell tumor	2.57E-03	FOXL2	1
atresia of oocytes	2.57E-03	FOXL2	1
fibrothecoma	2.57E-03	FOXL2	1
recurrent hydatidiform mole type 2	2.57E-03	KHDC3L	1

type 1 blepharophimosis, epicanthus inversus, and ptosis	2.57E-03	FOXL2	1
trophoblastic tumor	2.67E-03	KHDC3L,POU5F1	2
dysplasia of placental tissue	5.13E-03	H19	1
germ cell and embryonal neoplasm	1.31E-02	KHDC3L,mir-10,POU5F1	3
ovarian tumor	1.48E-02	DIRAS3,FOXL2,H19,HOXA5,MECOM,PRDM9	6
prostate cancer	1.62E-02	B4GALNT4,DIRAS3,MAD1L1,mir-10,mir-7,PRDM9	6
granulosa cell tumor	2.79E-02	FOXL2	1
hyperprolactinemia	3.53E-02	DRD4	1
ovarian cancer	4.22E-02	DIRAS3,H19,HOXA5,MECOM,PRDM9	5
